# Physical activity and its associated factors in females with type 2 diabetes in Riyadh, Saudi Arabia

**DOI:** 10.1371/journal.pone.0239905

**Published:** 2020-10-01

**Authors:** Badreldin Abdelrhman Mohamed, Mohamed Salih Mahfouz, Mohamed Farouk Badr

**Affiliations:** 1 Department of Community Health Sciences, College of Applied Medical Sciences, King Saud University, Riyadh, Saudi Arabia; 2 Department of Family and Community Medicine, Faculty of Medicine, Jazan University, Jazan, Saudi Arabia; 3 Nutrition and Food Science Department, Faculty of Home Economics, Helwan University, Cairo, Egypt; Suez Canal University Faculty of Medicine, EGYPT

## Abstract

Despite the benefits of physical activity (PA) for the management of type 2 diabetes Mellitus (T2DM), the topic of PA is poorly addressed in Saudi Arabia (SA), especially in females with T2DM. The present study examined PA and its associated factors in females with T2DM in Riyadh, Saudi Arabia. This observational cross-sectional study was performed in a random sample of 372 women with T2DM. A face-to-face interview that covered PA, health and environmental correlates of PA was performed. Discriminant analysis was used to determine which barriers had the greatest impact on PA in these women. The results showed that approximately 26.3% of the study participants met PA recommendations. Multivariate linear regression revealed lower levels of PA were associated with women who had more than three children (β = -0.17) compared to women with no children, older age (β = -0.18), women with a duration of diabetes ≥ 6 years (β = -0.16), women who were obese (β = -0.23), women with no family support (β = -0.20), no friend support (β = -0.13) and no healthcare provider support (β = -0.14). Discriminant analysis indicated that culture and tradition, lack of skills and knowledge, safety, fatigue, lack of time, weather conditions, and lack of facilities were the barriers that differentiated between the women who met and those who did not meet the PA recommendations. The present study suggests that the prevalence of PA is low and number of children, age, duration of diabetes, Obesity, family support, friend support and healthcare provider support are identified correlates of PA. These findings are valuable and should be used to design and implement future PA interventions, especially for women with T2DM. Healthcare providers may improve exercise levels and identify the specific barriers to reaching the recommended level of PA to improve health outcomes for each patient.

## Introduction

The Kingdom of Saudi Arabia (SA) has the second highest prevalence of diabetes in the Middle East, and it is ranked seventh in the world [1]. Diabetes mellitus (DM) is the fourth leading cause of death in SA, and it is the 65th leading cause worldwide, with a death rate of 35.61/100,000, which is a major public health problem [2, 3]. The number of individuals with diabetes worldwide increased to 422 million in 2014, in contrast to just 108 million in 1980, and the figures are expected to increase to 552 million by 2030, which will account for approximately 9.9% of the total world population [4]. The prevalence of type 2 diabetes mellitus (T2DM) in SA increased from 1.8% to 27.6% between 1998 and 2013 [5, 6]. If the disease follows the current trend, a prevalence rate of greater than 50% would be observed in adults by 2030 [7].

Much research supports the beneficial role of PA in the management of diabetes [8–10]. Despite the numerous health benefits of PA, its promotion is often inadequate, and a high rate of physical inactivity was reported in people living with diabetes [11–13]. Many studies in Western countries investigated factors associated with PA [14–16]. However, different attributes are likely present in Arabian countries. The traditional images of gender have prevented women from participating in PA. The social expectations for public behavior influence the choice of women’s active participation in any sport. Conservative social norms defining the traditional role of women influence the context in which women may be physically active. The cultural factors faced by Saudi females may limit exercise and decrease PA [17, 18].

The prevalence of PA in countries of the Gulf Cooperation Council (GCC) ranges from 26.5–28.4% in women [19]. One report suggested a high rate of physical inactivity in SA [20]. Physical inactivity in Saudi society is prevalent, and it ranges from a minimum of 43% in certain segments of society to 99% [20]. In contrast to developed countries such as the USA (35%) and UK (37%), the risk of inactivity was estimated at 44% in less developed countries [20]. A 2010 report by the World Health Organization (WHO) revealed that 74.9% of Saudi women were categorized as insufficiently active, which makes them the lowest prevalence of PA worldwide [21]. The perception in SA is that the females are housewives who generally engage in moderately intensive PA. However, they have the lowest prevalence of intensive and moderate PA (2%) worldwide.

Studies on PA in Saudi females are scarce. No previous study specifically assessed PA in SA females with T2DM. Leisure-time PA was the most frequently studied domain of physical activity [21–23]. In contrast, nonleisure PA, which is performed during one’s daily routine at home or work, was less studied in females in SA. The present study examined PA in females with T2DM to characterize the determinants of PA in the context of Saudi social and cultural influences.

## Materials and methods

### Design, setting and participants

An analytical cross-sectional study was carried out in the Diabetic Center at King Abdul Aziz University Hospital (KAUH), Riyadh, SA. This center is a leading center in the Kingdom of SA for the teaching and evaluation of diabetic patients, and it began to provide services in 1994. The study participants were Saudi females who attended the Diabetic Center at KAUH. Eligibility criteria included (1) age 18 years and older, (2) diagnosis of T2DM at least six months before the survey, and (3) the presence of a medical file at the center. T2DM women with severe conditions, such as stroke, pregnant women and patients with mental health issues were excluded.

### Sampling and data collection

A representative sample was calculated using the sampling formula for a single cross-sectional survey. Sample size was calculated depending on a 29% prevalence of T2DM in females in SA [24], 95% confidence interval, error not greater than 5%, and a nonresponse rate of 20%. The total sample size was 380 females. Subjects were selected randomly from the records of the Diabetic Center at KAUH. The data were collected using "face-to-face" interviews and a set of standardized questionnaires. Trained female students from the Community Health program, the College of Applied Medical Sciences, King Saud University interviewed the women.

### Study instruments

The following data were also collected: personal data, including age, education level, marital status, number of children, family income, duration of the disease, comorbidities, and number of cars in the household, and environmental factors, including proximity to parks and shopping centers. Social environmental factors included seeing females who exercise and knowing females who exercise. The physical environment included traffic, the presence of sidewalks, street lighting at night, intersections close to each other and having a house maid. Additional information on PA-related behaviors, such as social support, was included. Data on the most recent physiological measurements (within three months of the study) were extracted from the medical records. BMI was calculated as body weight (kg)/height (m2). Patients were classified according to the WHO classification as underweight (<18.5), normal (18.5–24.99), overweight (25–29.99) and obese ≥30. Prior to the main study, a pilot study was performed in the same center in a sample of 30 patients to test the study instrument. The patients involved in the pilot study were not included in the final study. The standardized questionnaires used involved the following items.

#### The International PA questionnaire

The International PA questionnaire (IPAQ) is a valid and reliable questionnaire that is used in different countries [25, 26]. The questionnaire was used to assess the PA of the participants at three specific levels of activity: walking, moderate and vigorous-intensity activities within each of the domains of work, transportation, domestic chores and leisure time and their frequency (days/week) and duration (minutes/day). To assess the recommended physical activity, we used the American Diabetes Association (ADA) recommendation that “adults with T2DM should engage in at least 150 minutes or more of moderate-to-vigorous intensity aerobic activity per week, spread over at least three days/week, with no more than two consecutive days without activity [27]. Shorter durations (minimum 75 minutes/week) of vigorous-intensity or interval training may be sufficient for younger and more physically fit individuals.” The new guidelines recommend moderate-intensity physical activity (i.e., 30 minutes of moderate-intensity physical activity ≥ five days/week).

#### Physician advice (PhA), sedentary behavior (SB) and social support (SS)

Physician advice for PA was defined as verbal or written messages provided by the physician, including recommendations, counseling, or written prescriptions to begin, maintain, or increase PA [28]. Physician advice was assessed via a direct question that was answered on a dichotomous scale: “In the last year has your physician given you advice to do any physical activities?” The responses were coded as 1 = yes or 2 = no. SB was self-assessed by participants using the Domain-Specific Sitting Time Questionnaire (D-SSTQ), which was validated for use in adults [29, 30]. The social support for exercise questionnaire developed by Sallis et al. [31], with 20 questions, was used to determine the amount of social support for exercise. Cronbach's α coefficients for family and friend support were 0.89 and 0.86, respectively. The questionnaire was also validated by Noroozi et al. [32] using exploratory and confirmation analyses.

#### Barriers to PA (BA)

The questionnaire on barriers was derived from several previous studies on PA [33–38]. Items that seemed appropriate for our questionnaire were extracted from related articles, and a primary questionnaire was designed. The content and face validity of the questionnaire were assessed by a panel of 8 experts from the Department of Community Health and Physical Education. Cronbach’s α was 0.78, the Spearman-Brown index was 0.81, and the stability was 0.79, which was assessed using the intraclass correlation coefficient. Participants were asked to assess how likely it was that each barrier affected their PA. This questionnaire rated each barrier on a 4-point Likert scale (very likely = 4, somewhat likely = 3, somewhat unlikely = 2, very unlikely = 1).

### Statistical methods

Descriptive and inferential statistics were used for data analyses. Data were described using means and standard deviation (S.D.) and frequencies and percentages for categorical variables. Chi-squared tests were used to determine the association of PA levels with participants’ characteristics. An independent sample t-test was used to analyze the difference between the two groups, meeting and not meeting PA recommendation. Multiple linear regression analysis was used with PA (Minutes per week) as the outcome variable. The assumptions of the linear regression models were evaluated. Our model controlled for possible confounding by the personal variables of age, education, income, marital status, duration of diabetes, and BMI and the environmental factors. For qualitative independent variables, a reference category was created. Discriminant analysis was used to determine which of the investigated variables made the greatest contribution between women with T2DM who met the PA recommendations and who did not meet the recommendations in Riyadh, Saudi Arabia. The level of statistical significance was set at *p-*value < 0.05. The data entry and analyses were performed using IBM Statistics SPSS version 20 (SPSS Inc, White Plains, NY, USA) software.

### Ethical approval

This study was performed in accordance with ethical standards within the political borders of the Kingdom of Saudi Arabia. All participants involved in this study read, understood and signed a written consent form. The ethical committee at the College of Applied Medical Sciences, King Saud University, Saudi Arabia approved the study.

## Results

The study sample was initially 380 females, but eight respondents refused to be included in the study. Therefore, 372 women (97.9%) completed the study. Table 1 shows some selected characteristics and the prevalence of meeting PA recommendations among the participants. Most women were married (78%) and aged between 50–69 years (64%), with a mean age of 57.3 ± 9.8 years. Most females were diabetic for more than three years (77.7%). Approximately 85% of the respondents were overweight or obese, and 41.4% were classified as obese (mean BMI was 36.1 ± 5.6 kg/m2). The overall prevalence of meeting PA recommendations in the women was 26.3% [95% CI: 22.1–31.1] and was significantly higher for single women 46.3% than married women 20.7% (***P*** = 0.021). Meeting PA recommendation also decreased significantly with increasing age (***P*** = 0.026). The prevalence of meeting PA recommendations differed significantly according to children ever born, educational level and family income (***P <*** 0.05 for all).

**Table 1:** Selected socioeconomic characteristics of the women and the prevalence of meeting physical activity recommendations.

Variable	N (%)	Meeting PA recommendation*	*p*-value
**Marital status**			0.021
Married	290 (78)	60 (20.7)	
Single	82 (22)	38 (46.3)	
**Children ever born**			0.042
0	78 (21.0)	33 (42.3)	
1–3	141 (37.9)	42 (29.8)	
>3	153 (41.1)	23 (23.5)	
**Age group (years)**			0.026
< 40	33 (8.9)	16 (48.5)	
40–49	78 (21)	32 (41.0)	
50–59	134 (36)	23 (17.2)	
60–69	104 (28)	17 (15.4)	
≥ 70	23 (6.1)	2 (8.7)	
**Education level**			0.027
Illiterate	77 (20.7)	10 (13)	
Primary and intermediate	122 (32.8)	29 (23.8)	
Secondary	150 (40.3)	45 (30.0)	
University and above	24 (6.5)	14 (58.3)	
**Occupation**			0.624
Housewife	257 (69.1)	71 (27.6)	
Working	115 (30.9)	37 (23.4)	
**Family Income (SR)**			0.036
< 5000	131 (35.2)	23 (17.6)	
5000 –less than 9,000	141 (43.2)	39 (27.7)	
9,000–13,000	62 (16.8)	21 (33.9)	
More than 13,000	38 (4.9)	15 (39.5)	
**Number of cars**			0.433
1	108 (29.0)	30 (27.8)	
2	160 (43.0)	45 (28.1)	
>2	104 (28.0)	33 (31.7)	
**Overall Prevalence of meeting PA**	**372 (100)**	**98 (26.3)**	**95% C.I. [22.1–31.1]**

*Based on the American Diabetes Association (ADA).

C. I. Confidence Interval., p-value based on chi-squared test

Table 2 lists some environmental and health characteristics related to the participants. Most of the women described traffic in Riyadh as heavy (90.9%) and indicated that the streets had sidewalks (48.1%). Most of the women (90%) reported that they did not see other females exercising in the neighborhood or did not know other females who exercised, that they lived far from parks (75.5%) and that intersections in the streets were close to each other (82.5%). Meeting PA recommendation was more common in females with no comorbidities, with a shorter duration of diabetes, who see other females who exercise, who have a house maid and who live where streets have sidewalks.

**Table 2:** Selected environmental and health characteristics of the participants and the prevalence of meeting the PA recommendations.

Variable	N (%)	Meeting PA recommendation*	*p*-value^1^
**Proximity to parks**			0.045
Very close	17 (4.6)	7 (41.2)	
Kind of close	74 (19.9	29 (39.2)	
Far	281 (75.5)	91 (32.4)	
**Know females who exercise**			0.621
Yes	57 (15.3)	28 (49.1)	
No	315 (84.7)	149 (47.3)	
**See females who exercise**			0.028
Yes	81 (21.5)	47 (58.0)	
No	292 (78.5)	71 (24.3)	
**Physical environment**			0.037
Traffic			
Light	23 (3.5)	11 (47.8)	
Moderate	31 (5.6)	13 (41.9)	
Heavy	318 (90.9)	74 (23.3)	
**Presence of sidewalks**			0.024
Yes	179 (48.1)	73 (40.8)	
No	193 (51.9)	25 (13.0)	
**Street lighting at night**			0.831
Yes	234 (89.8)	112 (30.1)	
No	38 (10.2)	11 (28.9)	
**Intersections close to each other**			0.032
Yes	64 (17.5)	16 (25.0)	
No	307 (82.5)	42 (13.7)	
**Body mass index (BMI) (kg/m**^**2**^**)**			0.014
18.5–24.9 (normal)	62 (16.7)	18 (29.0)	
25–29.9 (overweight)	156 ((41.9)	34 (21.8)	
> 30 (obese)	154 (41.4)	23 (14.9)	
**Comorbidities**			0.025
Yes	207 (82.8)	37 (17.9)	
No	165 (17.2)	51 (30.9)	
**Duration of diabetes (years)**			0.001
Less than 3	85 (22.8)	44 (51.8)	
3–6	132 (31.7)	36 (30.5)	
More than 6	155 (41.7)	18 (11.6)	
**Have a housemaid**			0.057
Yes	107 (28.8)	48 (44.9)	
No	256 (71.2)	76 (29.7)	
**Support from physician**			0.006
Yes	71 (19.1)	27 (38.0)	
No	301 (80.9)	22 (7.3)	
**Other PA continuous measures**			
Measure	**Meeting PA Mean (SD)**	**Not-Meeting PA Mean (SD)**	***p*-value**^**2**^
**Social Support**			
Support from family	21.1 (2.3)	12.8 ±1.6	0.001
Support from friends	5.7 (0.81)	2.9 ±0.65	0.001
**Sedentary behavior**			
TV viewing (hrs/week)	6.7 ±1.8	9.1± 1.6	0.001
Sitting (min/day)	689 ±40.6	806 ±28.4	0.001

*Based on the American Diabetes Association (ADA).

p-value^1^ based on chi-squared test; p-value^2^ Based on independent sample t test

Table 3 presents the results of the multiple linear regression model. The model found that lower levels of PA were associated with women who had more than three children (β = -0.17) compared to women with no children, older age (β = -0.18), women with a duration of diabetes ≥ 6 years (β = -0.16), women who were obese (β = -0.23), women with no family support (β = -0.20), no friend support (β = -0.13) and no healthcare provider support (β = -0.14), women who spent more time sitting (β = -0.20) and watching TV (β = -0.23) and women in areas with heavy traffic.

**Table 3:** Correlates of physical activity in Saudi women with T2DM.

Variable	B	SE	β	*p-*value
**Marital status**				
Single (Ref: married)	4.41	1.74	0.13	0.03
**Number of children**				
Ref (no children)				
1–3	-2.62	1.89	-0.07	0.42
> 3	-4.07	1.23	-0.17	< 0.01
**Age (years)**	-4.52	1.28	-0.18	< 0.01
**Education level**				
Ref (Illiterate)				
Primary and intermediate	2.73	1.85	0.08	0.69
Secondary	3.46	2.78	0.06	0.51
University and above	9.68	3.04	0.17	< 0.01
**Duration of diabetes (years)**				
Ref (Less than 3)				
3–6	-1.63	1.08	-0.07	0.08
More than 6	-3.81	1.25	-0.16	< 0.01
**Family income (SR)**				
Ref (less than 5000)				
5000 –less than 9,000	1.64	1.23	0.07	0.46
9,000–13,000	1.94	1.84	0.05	0.52
More than 13,000	3.07	0.79	0.20	< 0.01
**Body mass index BMI (kg/m**^**2**^**)**	-4.22	0.97	-0.23	< 0.01
**Sedentary behavior**				
TV viewing (hrs/week)	-2.87	0.74	-0.23	< 0.01
Sitting (min/day)	-3.14	0.88	-0.20	< 0.01
**Proximity to parks**				
Ref (Far)				
close or kind of close	2.68	1.07	0.13	0.03
**Traffic**				
Ref (light, moderate)				
Heavy	-3.88	1.04	-0.19	< 0.01
**See females who exercise**				
Yes (Ref: No)	2.88	1.09	0.14	0.01
**Social support**				
Family support (Ref: Yes)	-3.72	0.98	-0.20	< 0.01
Friends support(Ref: Yes)	-2.44	0.13	-0.13	< 0.01
Physician support(Ref: Yes)	-2.03	0.14	-0.14	0.02

B: unstandardized regression coefficient; SE: standard error for B

β: standardized regression coefficient.

Table 4 lists the discriminant function structure matrix. The discriminant analysis produced a statistically significant Wilks’ λ = 0.81, χ2 = 138.3 (***P*** < 0.001). The discriminant analysis identified culture and tradition reasons, lack of skills and knowledge, fatigue, safety, lack of time, weather conditions and lack of local facilities as statistically significant (***P*** < 0.01) discriminators of meeting the recommendations. The assumption of equal covariance matrices was tested. The Box’s M test was not statistically significant (***P*** = 0.187), which suggests that the covariance matrices were equal. The model correctly classified 71% of females included in the sample. Specifically, the model correctly classified 65.4% of women who met the PA recommendations and 77% of women who did not meet the recommendations. The discriminant efficiency was 76.2%. The barriers were arranged in order of their contribution to the discrimination between women who met the recommendations and women who did not meet the recommendations.

**Table 4:** Discriminant function structure matrix.

Reason	Function loading
Culture and tradition *	0.762
Lack of skills and knowledge*	0.728
Fatigue*	0.704
Safety*	0.681
Lack of time*	0.649
Weather conditions*	0.611
Lack of local facilities*	0.604
Fear of injury/fall	0.421
Lack of resources	0.386
Lack of motivation	0.276
Lack of interest	0.207
Laziness	0.174

*p-value < 0.01.

## Discussion

The current study revealed that PA levels were low in SA diabetic patients, and only 26.4% of the participants were active. This low prevalence of PA may be explained by the facts that the general population from which the sample was selected was equally physically inactive [20], and women in the Arabian culture are likely to take part in light-to-moderate intensity activities. There is a high probability of recall error, and error in measurement is greater for light-to-moderate intensity activities, which would ultimately lead to an underestimation of overall physical activity level [39]. Tessa found that women had difficulty recalling the time they spent in different activities due to the overlapping of activities [40].

The prevalence in the current study was higher than that in Al-Kaabi et al [41] in the United Arab Emirates (11%), Alghafri et al [42] in Oman (12%), Siba et al [43] in Lebanon (9%) and Abraham et al in Ethiopia (11%) [44]. However, the prevalence was lower than previous studies in patients with T2DM in the USA [45] (39%), Brazil (30.7%) [46], Nigeria [47] (40.2%), Nepal (41%) [48] and Malaysia (31.9%) [49]. Of greatest concern is the fact that 28.7% of participants in our study reported no activity (MET = 0), which is lower than Oman [50] (60.3%) and the USA (47.4%) [51]. However, these comparisons between countries must be interpreted with caution and consider the actual meaning of physical activity and the characteristics and culture of the study population [52, 53].

Our findings should be evaluated in the context of Saudi culture and tradition and the role of women in this culture. Cultural norms and social expectations require that women do not exercise in mixed gender settings, which reduces existing opportunities for women to be involved in any PA [19].

Fatigue and tiredness from the disease are major factors in the lack of motivation to engage in leisure-time physical activity among Saudi women. Cynthia et al. [54] stated that alterations in levels of blood glucose caused fatigue in diabetic individuals, and this effect may eventually result in hyperglycemia or blood glucose fluctuations. Findings on the relationship between A1c and fatigue are mixed. Our data did not support any relationship between A1c and fatigue, which is consistent with other studies of T2DM in which only very minor or no association between A1c and fatigue was found [55, 56].

Riyadh has the highest number of casualties and accidents in SA. A total of 91% of participants reported heavy traffic, 82.5% reported intersections that were not close to each other, and 51.9% indicated the absence of sidewalks. Sadly, approximately one third (29%) of people who were injured or died due to accidents in the country resided in the Riyadh province [57].

Lack of time was cited as a barrier to PA in populations with diabetes [35, 37, 58, 59]. For Saudi women, multiple role responsibilities may severely limit the time allotted for leisure-time physical activity. For unemployed women, domestic duties around the house, such as cleaning, looking after children or grandchildren and preparing food, limited their time to participate in any PA.

A women's decision to participate in physical activities and exercise is also related to the availability and accessibility of recreational facilities. Most neighborhoods in Riyadh fail to provide accessible facilities for recreational activities, such as walking paths, to support PA among its citizens [6]. The norm here is to walk to have an active lifestyle, which is also the cheapest form of PA. A healthy physical environment influences PA behavior and may be achieved by providing safe recreational facilities and transit options. Therefore, interventions and development regulations at the level of policy-makers will surely help overcome this problem.

A few patients also reported the adverse climate of the region as a hindrance in performing physical activities. The climate in SA is characterized by a hot, arid climate, where daytime temperatures may rise to 45°C in summer and plummet below zero in winter, which are nonconducive environments to PA. Serour et al and Thomas et al [60, 61] showed that major barriers to exercise in patients with type 2 diabetes were firstly poor climate and secondly hot summers.

Many studies showed the importance of social support in enhancing PA, especially support from family, friends and physicians [62, 63]. Our study observed a small nonsignificant relationship for family support and PA, which is consistent with other studies that measured social support [64], but contradicted Melanie et al [15], who found a positive significant correlation between family support and PA. Women in SA do not receive the same kind of encouragement as their male counterparts to be socially independent and physically active in sports activities. Women are constrained by a culture that does not condone their involvement in PA, and the non-approval of their participation in PA by family members due to demands of domestic responsibilities coupled with religious factors may be due to a diminished awareness of the impact of physical activity on the improvement of the status of diabetic patients in individuals and families.

The time since diagnosis is a possible confounding variable that could affect the physical activity behavior of diabetic individuals. Shanti and Arja [48] observed that higher MET values were attained by individuals who were diagnosed with diabetes between 6 months to two years compared to patients who were diagnosed 6 months before the study or more than 2 years prior at the time of the study. Most of the women in our study (64.7%) were diagnosed with diabetes at least four years prior. A total of 56% reported being more inactive than women who were diagnosed for less than one year. Plotnikoff [65] reported the same result but only considered the time of diagnosis of less than one year.

Most women do not know their activity potential because of the lack of exposure to any kind of PA. The idea of exercise is foreign in the Arab culture, particularly for women. Cristina et al [66] found that Arabic-speaking women in Australia associated the increase in heart rate, sweating and breathlessness to illness, when these changes were actually due to PA. An uncertainty related to safe levels of activity is persistent in women, which is primarily due to the absence of any sports activities in female schools. Previous results demonstrated that a good predictor of physical activity in later life was attributed to participation in sports during adolescence and childhood. If the involvement in physical activity during youth was continuous, then there was an increased potential for PA in adulthood [67, 68]. Our data revealed a significant negative association between BMI and PA. In an independent study describing PA patterns in French adults [69], researchers found that women with increased BMI demonstrated a decreased PA by 1.31–1.67 MET minutes/week. Pearte et al [70] also reported that BMI was related to a lower level of physical activity and fewer blocks walked per week.

Our results showed that adults aged 60 years and older had a higher level of physical inactivity than younger adults. This result is similar to previous findings that reported that physical inactivity increased with advancing age [71, 72]. One possible explanation is that participants in this age group are hesitant to participate in physical activity due to several reasons, including lack of interest, physical symptoms (for example, shortness of breath, joint pain, lack of energy), difficulties with access, and a lower self-efficacy of physical activity [73, 74].

There are limited data in the literature that connect marital status with physical activity. We observed that participants who were unmarried were more likely to be physically active than married participants. This finding is consistent with AlNozha et al [75] in Saudi Arabia, who found that single individuals were physically more active, possibly because single women have more leisure time, fewer family responsibilities and are not yet confined by life stressors. Previous research showed that mothers with children were less active than women without the responsibility of caring for a child [76, 77]. However, the literature is mixed on the influence of the age of mother and the number of children at home as predictors of the physical activity of mothers [78, 79]. The age of children was significantly related to PA. Mothers with younger children were less physically active than mothers with older children. Our results showed that motherhood negatively affected women’s participation in PA. Women with more than three children were less likely to be physically active than women with no children. In Saudi culture, gender inequality is still experienced in matters related to household management, and the effect is greater in families with young children. Therefore, PA in women has a low priority status, and women are unlikely to be able to negotiate the time and resources needed to maintain PA. A large mean family size was also attributed to decreased physical activity because of less time to spend on these activities [80, 81]. The cultural norms for women, including cooking and childcare, are also noteworthy.

### Limitations

The present study was performed in Riyadh, which decreases the generalization of the results to other regions or populations, for example, adults in rural areas. Second, data were obtained using a self-reported questionnaire, which is a source of potential error. Participants may underestimate or overestimate their physical activity behavior, which may affect the study findings. Tessa and Cornelia found that women had difficulty recalling the time they spent in different activities, which was primarily due to activities overlapping [40]. Third, due to the cross-sectional nature of the study, the temporality of certain associations cannot be established with confidence. Nevertheless, it clearly snapshots the current situation and may help guide the development of PA promotional interventions and programs for T2DM individuals via profiling populations who are least active. Fourth, the lack of comparable data in SA limited the extent of the analyses in this study. Despite these limitations, the data make an important contribution to the body of literature in Saudi Arabia by examining the factors that influence the physical activity levels of diabetic Saudi women.

## Conclusion

The present study is the first study to provide relevant data on the variables influencing PA in female patients with T2DM in Riyadh, SA. The prevalence of PA was low. The key population demographic subgroups with a low prevalence of PA were women who were aged ≥50 years, married and with children and had a higher BMI. Culture and tradition, fatigue, safety, comorbidities, and fear of injury were the main significant barriers to PA.

To promote PA, we recommend that mass media organize an educational program that encourages female participation in PA. There is a need to create an enabling environment by establishing large public parks for women equipped with sports facilities and trails for walking. Future research should be directed towards examining how various physical activities affect a wider range of females in Riyadh and the whole country.
